# Novel design and development of a 3D-printed conformal superficial brachytherapy device for the treatment of non-melanoma skin cancer and keloids

**DOI:** 10.1186/s41205-019-0045-z

**Published:** 2019-07-22

**Authors:** Jennifer Chmura, Arthur Erdman, Eric Ehler, Jessica Lawrence, Christopher T. Wilke, Brent Rogers, Clara Ferreira

**Affiliations:** 1 Biomedical Engineering Department, University of Minnesota, Minneapolis, MN USA; 2 Medical Devices Center, University of Minnesota, Minneapolis, MN USA; 3 Department of Radiation Oncology, University of Minnesota Medical School, Minneapolis, MN USA; 4 Department of Veterinary Clinical Sciences, University of Minnesota, St Paul, MN USA

**Keywords:** 3D printing, Brachytherapy, Radiation therapy, Superficial

## Abstract

**Background:**

Skin tumors are the most predominant form of cancer in the United States. Radiation therapy, particularly high dose-rate (HDR) brachytherapy, provides an effective form of cancer control when surgery is not possible or when surgical margins are incomplete. The treatment of superficial skin cancers on irregular surfaces, such as the nose, lips or ears, present challenges for treatment. To address this issue, we designed and constructed a novel conformal superficial brachytherapy (CSBT) device prototype to improve patient-specific treatment for complex sites. The device is mounted on an automated remote after-loader, providing limited radiation exposure to operating personnel, is inexpensive to construct, and offers a unique method of conformal surface radiation therapy.

**Results:**

A prototype of the CSBT device was successfully manufactured. A computed tomography (CT) scan of a Rando phantom was used to plan the target treatment area. The CSBT device has a hexagonal lattice array of retractable rods with radioactive seeds placed at the tip of each rod. A 3D-printed conformal shape insert with a hexagonal array of cylindrical projections of varying length is driven into the rods by a single linear actuator. The rods are displaced to conform to the patient’s skin. This elegant device design permits the delivery of radiation to complex targets using readily available beta-emitting radionuclides, such as Yttrium-90 (Y-90) or Strontium-90 (Sr-90).

**Conclusion:**

A working prototype of a novel CSBT device was built using 3D-printing technology that provides a safe and economically attractive means of improving radiation delivery to complex treatment sites.

**Electronic supplementary material:**

The online version of this article (10.1186/s41205-019-0045-z) contains supplementary material, which is available to authorized users.

## Background

Three-dimensional (3D) printing has garnered tremendous interest amongst medical professionals in recent years. 3D printing not only offers customizable printing but also offers a variety of materials in which to investigate or promote rapid technological advancement for patients. It is estimated that 5.4 million new cases of non-melanoma skin cancer were diagnosed in the United States in 2012 alone [1]. Non-melanoma skin cancer includes basal cell carcinoma (BCC), squamous cell carcinoma (SCC), and non-epithelial skin cancer. Current treatment modalities for non-melanoma skin cancers include Mohs micrographic surgery and radiation therapy (RT), which includes external electron beam, skin brachytherapy and electronic brachytherapy [2–4]. RT is an effective primary treatment for non-melanoma skin cancer with recurrence-free rates exceeding 90%, even for tumors that recur following surgery [5–7]. RT is currently used routinely as adjuvant therapy following surgery for improved local control, particularly in cases at high-risk for postoperative recurrence [8–11]. For patients in which surgery is contraindicated, or in which disfigurement or postoperative scarring are likely, RT alone is the preferred treatment method [10–12]. Indeed, a recent position statement by the American Academy of Dermatology recommends superficial RT as the most appropriate second line option in cases in which surgery is contraindicated or has been declined, however additional research on surface brachytherapy is needed [13]. A primary limitation of current methods of radiation delivery include failure to deliver adequate and homogeneous dose coverage to small or irregular fields, such as facial tumor sites. Brachytherapy, or short distance RT delivered with the use of radioisotopes, has improved the ability to treat smaller skin fields, but do not conform well to uneven surfaces, such as the eyes, lips, and nose [14]. This may result in the inclusion of additional normal tissue in the radiation field to ensure good dose is administered to tumor, which can increase radiation toxicity and long-term cosmesis. Therefore, there is a clear need to develop more conformal brachytherapy modalities that are suitable for a wide range of tumor shapes and diameters to fit various anatomic sites. The primary objective in the design of the conformal brachytherapy device for clinical use presented herein was to improve patient-specificity by creating a device that delivers a uniform dose of radiation to irregular curved surfaces. The ability to balance treatment efficacy and normal tissue complications is of fundamental importance in radiation oncology and an adjustable brachytherapy device that uniquely conforms to each patient is necessary, given wide variation in human anatomy.

**Fig. 1:**
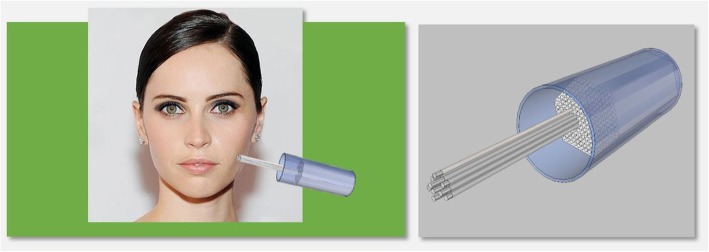
Conformal superficial brachytherapy applicator model

In this work we describe the mechanism of action of a novel conformal superficial brachytherapy (CSBT) applicator (Fig. 1). The device concept was previously described and we have adapted this work using 3D-printing techniques to simplify the design and develop a functional prototype [15]. The aim was to create a device that met the following characteristics: i) capable of conforming to small and irregular or complex skin or body surfaces not amenable to external electron beam therapy, ii) individualized to allow patient-specific modifications, iii) safely handled while loaded with different radionuclides, iv) mountable on an automated remote after-loader to limit personnel exposure to radiation, and (iv) reusable.

## Methods

### Determination of the treatment anatomy

In order to test the conformal ability of the device on an irregular surface such as a human nose, an anthropomorphic Rando phantom was used. This phantom models the routine diagnostic computed tomography (CT) images that are routinely obtained for radiation treatment planning. A DICOM dataset of Rando phantom was constructed into an STL surface representation file using the program 3D Slicer4 open source medical imaging software platform.

**Fig. 2:**
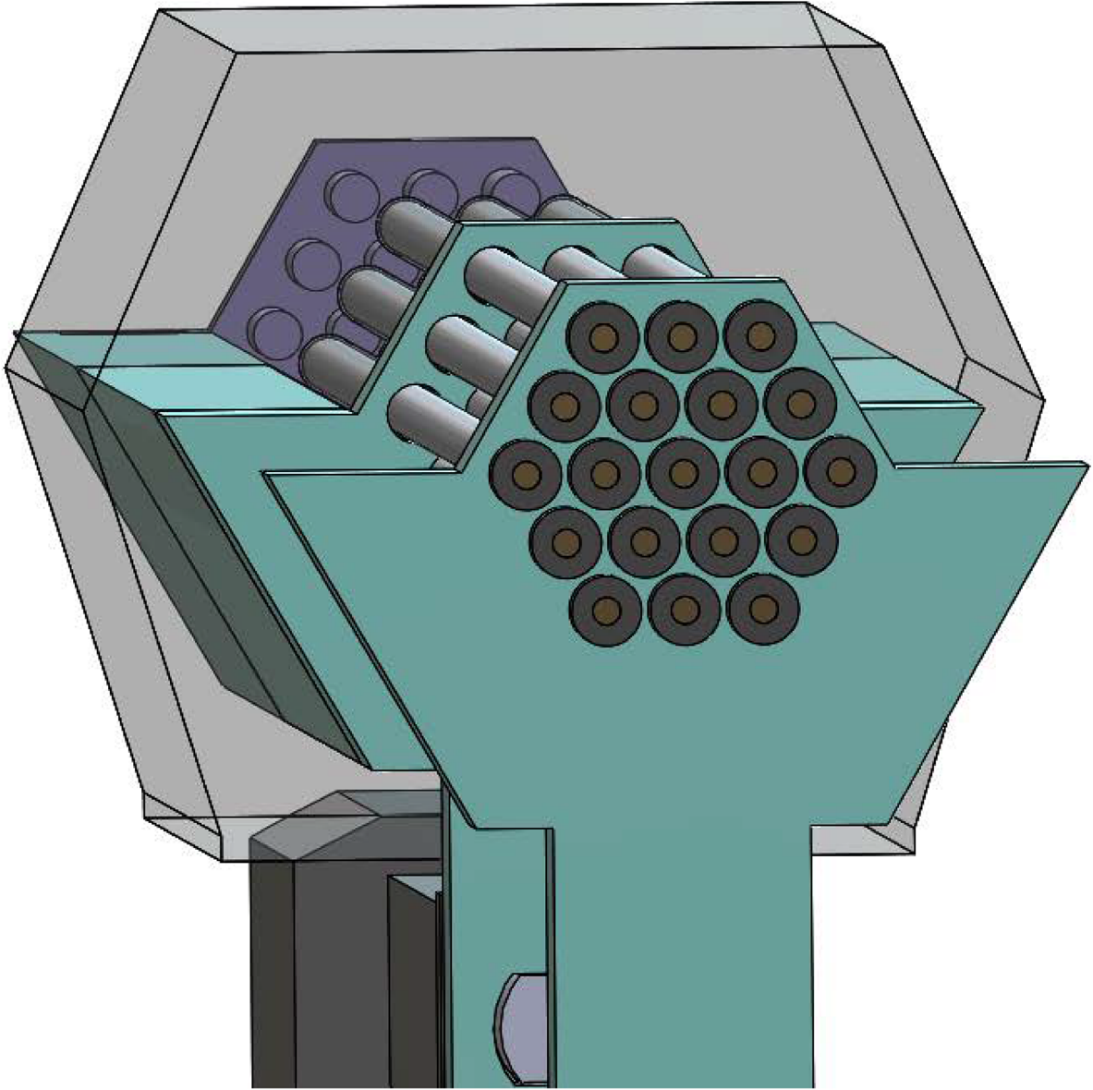
Hexagonal array of rods

A hexagonal lattice array with equally spaced (4.50 mm distance) elements was arranged perpendicular to the target treatment area (Fig. 2). The closest element of the array was placed at a distance of 0.3 cm from the skin, based on results from preliminary simulations showing the best dose conformity at this distance. At each element of the array, the CSBT device has a retractable rod with a radioactive seed placed at the tip of each rod. The rods are able to move independently of one another so that each rod projection can conform to different projections of the skin surface of a patient.

The projection of the array onto the skin at each element was used to determine the rod displacement. The length of each of the cylindrical rods on the hexagonal lattice array of the 3D-printed conformal shape was matched to the rod displacement calculated with the array such that each rod in the array would be 0.3 cm from the skin. In practice, the rods would not all be the same distance from the skin. A Monte Carlo simulation of several rods of the device and their distance to the skin would instead be used to determine how best to arrange the rods so that the dose is applied to the target volume while sparing the volume outside of that target.

### 3D printing

The working prototype of the device was completed by 3D printing the components modeled in Solidworks 2017 (Dassault Systemes, Waltham, MA) on either the Monoprice IIIP (Monoprice, Inc., Brea, CA; build volume 120 × 120 × 120 mm) or the Creality CR-10 (Shenzhen Creality 3D Technology Co., Ltd., Shenzhen, China; volume 300 × 300 × 400 mm) 3D printers using readily available polylactic acid (PLA) filament. The components were assembled together using M4 screws. The tips of the device were printed using the Form 2 SLA (Formlabs, Inc., Somerville, MA) 3D printer. The rubber ring and the rubber seal were joined to the metal rods using 3 M 08008 Black Super glue.

## Results

### Design and fabrication of the prototype CSBT device

The design of the CSBT device is elegant, simple to construct, clinically viable, and inexpensive. Moreover, components can be made with commercially available plastic material, with the overall construction sufficiently safe to allow testing with radioactive seeds. While the device is at rest, an aperture seals the end so that the device can be handled without exposure to radiation. Furthermore, the device is controlled electronically, allowing technicians and nurses associated with the brachytherapy procedure to be distant from the device to limit radiation exposure.

### Device components

**Fig. 3:**
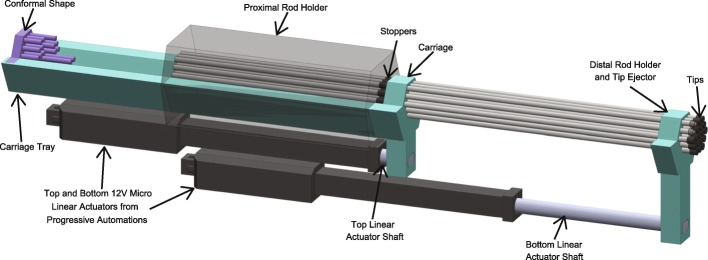
Internal mechanism of the device

The internal structure of the device is shown in Fig. 3. The proximal rod barrel and the linear actuators are stationary and fixed to the casing of the device. All other parts may move along the z-axis but not in any other direction. The shafts of the linear actuators are fastened to the carriage and to the tip ejector using screws. The casing of the device surrounds the shape of the carriage and tip ejector to form a track, so that these parts can be guided linearly by the actuators. The casing provides high torsional stability and torque load capacity for these parts. The conformal shape can be removed or placed into the device and is held in place by a screw.

**Fig. 4:**
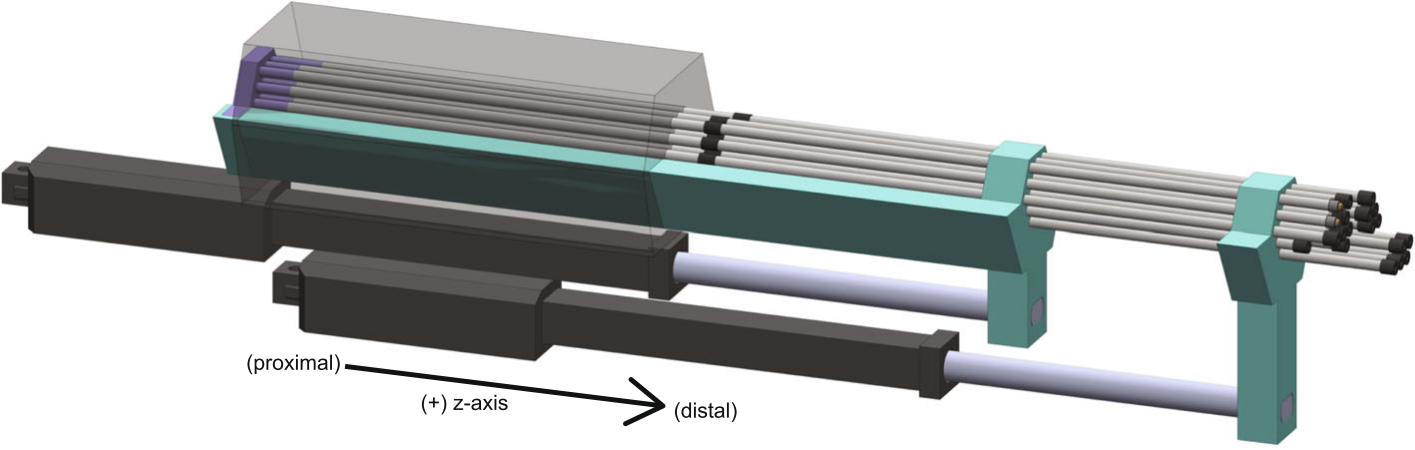
ZY-axis view of the device

Additionally, an array of metal rods sits in the device. The proximal end of the tungsten carbide rods sits in the proximal rod barrel and is held there by friction. In the center of the rods is a stopper situated between the proximal rod barrel and the carriage (Fig. 4). At rest, the stopper is held in place and prevents the rods from moving. The conformal shape is placed on the tray of the carriage and fixed in place with a screw.

The action of the device is shown in the animation in Additional file 1. As the top linear actuator is moved forward along the z-axis, the carriage moves forward and no longer restrains the movement of the rods at the stopper. However, friction at the proximal rod barrel prevents linear movement of the rods. The multiple cylindrical projections of the conformal shape fit within the cylindrical barrels of the proximal rod barrel and **provides a force that overcomes the friction between the rods and the proximal rod barrel. The rods are then displaced a set distance according to the length of the conformal shape’s projections. After a designated, pre-determined treatment time, the top linear actuator retracts; the carriage catches the stopper on the rods, causing the rods to retract to their original position**.

### Loading the tips of the device

**The tip loading plate has cutouts in the same hexagonal array as the device. The tips are placed inside the cutouts with their proximal ends facing upwards. The activated cylindrical radioisotopes of Ytrium-90 are easily transferred from their containment vial to the inside of the tips with various instruments. A glass aspirating pipette attached to a vacuum or forceps can also be used**.

**Fig. 5:**
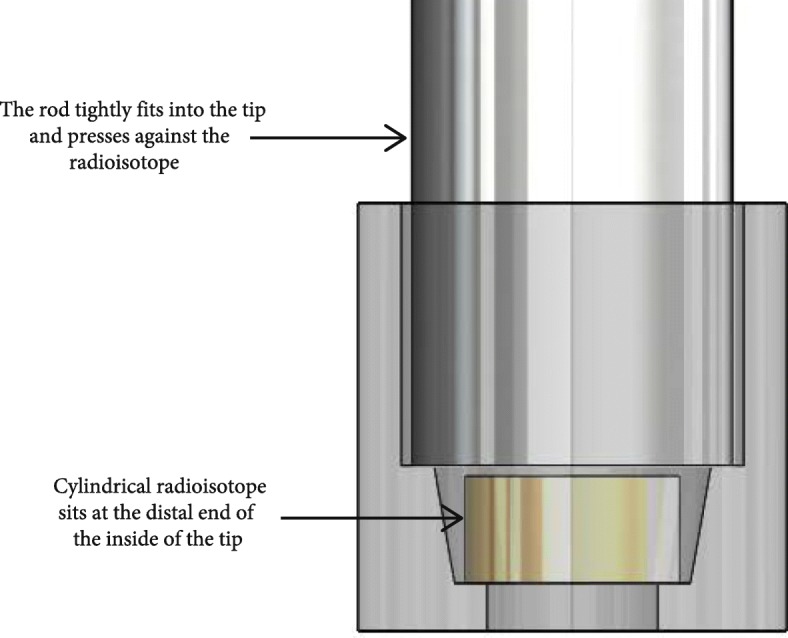
Design of the device tips

**The tips of the device (Fig. **5**) are specially designed to work** with cylindrical radioisotopes. When the cylindrical radioisotope is dropped into the tip, an internal taper within the tip helps orient the radioisotope so that the cylindrical base approximately sits against inside of the tip. Later, when the rods are fit into the proximal end of the tip, the rods correct the orientation of the radioisotope and ensure the base of the radioisotope sits flush against the inside of the tip.

**Fig. 6:**
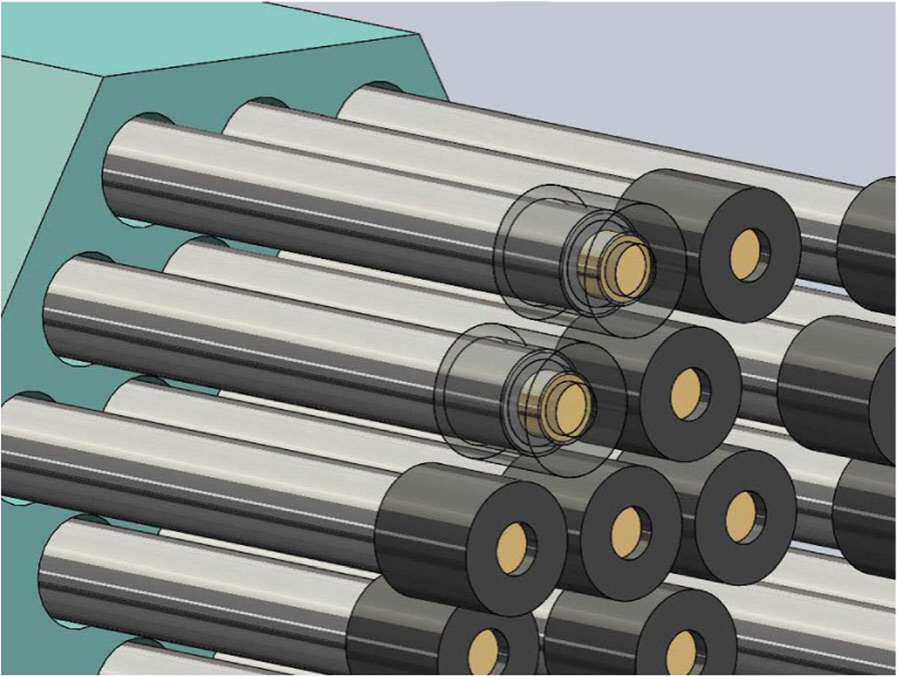
Tips loaded on the distal end of the rods

An animation showing how the tips are loaded onto the device is shown in Fig. 6.

### Placement of the applicator

The device holder is a Mount It! Articulating Arm (Mount It! San Diego, CA) for computer monitors and it allows both rotation and translation for accurate positioning and stability of the applicator. The patient-specific conformal shape is loaded at the proximal end of the device. The rod guides are multiple cylindrical projections of various lengths protruding out of the conformal shape that are arranged in the hexagonal array. The lengths of the rod guides represent the planned treatment geometry of the radioactive seeds on the patient’s skin surface. The conformal shape can be 3D printed by the clinician prior to treatment to increase efficiency of the treatment.

### Unloading the tips of the device

To unload the tips, the distal end of the device is again placed into the tray. The lower linear actuator is moved forward, and the distal rod holder overcomes the friction holding the tips to the rods. The tips are displaced into the tray for disposal.

## Discussion

The increasing availability and versatility of 3D printing has vastly changed the landscape of medical technology development. Coupled with the opportunities created to develop affordable and customized parts using 3D printing, there is a trend in medicine to provide personalized treatment for improved care and outcome measures. Individualized, customizable devices like the CSBT described here for cancer patients, offers an opportunity to improve radiation dose distribution to a target volume while reducing the amount of normal tissue that is irradiated. The CBST we designed permits the superposition of small treatment fields at the skin surface so that patient- and tumor-specific dosing plans can be altered to best suit the neoplastic lesion needing treatment.

Importantly, the device described here may significantly lower the overall cost of treatment compared to conventional electron therapy. By improving dose distribution and tumor control, there is also the possibilty of reducing patient visits over time by preserving cosmesis and reducing risk of tumor recurrence. While it does require the purchase of a reliable printer, the design of a device such as this may be widely implemented across radiation institutions once it is validated for clinical use. The long-term goal of work such as this is to improve the ability to target irregular skin lesions, particularly those over sites like the nose or ears, by taking advantage of 3D printing technology. A successful, clinical device will expand affordable care options to many skin cancer patients, and improve patient comfort and compliance.

## Conclusion

In summary, we have designed, created, and tested a novel device that provides patient-specific treatment for non-melanoma skin cancer lesions, especially in difficult treatment areas or irregular tumors. The working prototype of the device will be used in the future for film dosimetry experiments and animal experiments to test the radiation dose distribution once radioactive seeds are loaded to the device.

## Additional file

Additional file 1: Video of the action of the device. (AVI 3267 kb)

## Abbreviations

3D: Three-dimensional
BCC: Basal cell carcinoma
CSBT: Conformal superficial brachytherapy
CT: Computed tomography
HDR: High-dose-rate
SCC: Squamous cell carcinoma
Sr-90: Strontium-90
Y-90: Yttrium-90

## Authors’ information

The authors include researchers and students from the University of Minnesota.
